# Does Plant Origin Influence the Fitness Impact of Flower Damage? A Meta-Analysis

**DOI:** 10.1371/journal.pone.0146437

**Published:** 2016-01-19

**Authors:** Catalina González-Browne, Maureen M. Murúa, Luis Navarro, Rodrigo Medel

**Affiliations:** 1 Departamento de Ciencias Ecológicas, Facultad de Ciencias, Universidad de Chile, Las Palmeras 3425, Santiago, Chile; 2 Departamento de Biología Vegetal, Universidad de Vigo, Campus Lagoas-Marcosende, 36200, Vigo, Spain; Helmholtz Centre for Environmental Research - UFZ, GERMANY

## Abstract

Herbivory has been long considered an important component of plant-animal interactions that influences the success of invasive species in novel habitats. One of the most important hypotheses linking herbivory and invasion processes is the enemy-release hypothesis, in which exotic plants are hypothesized to suffer less herbivory and fitness-costs in their novel ranges as they leave behind their enemies in the original range. Most evidence, however, comes from studies on leaf herbivory, and the importance of flower herbivory for the invasion process remains largely unknown. Here we present the results of a meta-analysis of the impact of flower herbivory on plant reproductive success, using as moderators the type of damage caused by floral herbivores and the residence status of the plant species. We found 51 papers that fulfilled our criteria. We also included 60 records from unpublished data of the laboratory, gathering a total of 143 case studies. The effects of florivory and nectar robbing were both negative on plant fitness. The methodology employed in studies of flower herbivory influenced substantially the outcome of flower damage. Experiments using natural herbivory imposed a higher fitness cost than simulated herbivory, such as clipping and petal removal, indicating that studies using artificial herbivory as surrogates of natural herbivory underestimate the real fitness impact of flower herbivory. Although the fitness cost of floral herbivory was high both in native and exotic plant species, floral herbivores had a three-fold stronger fitness impact on exotic than native plants, contravening a critical element of the enemy-release hypothesis. Our results suggest a critical but largely unrecognized role of floral herbivores in preventing the spread of introduced species into newly colonized areas.

## Introduction

Research on flower herbivory has grown remarkably over the past few decades, which has permitted to confirm its prevalence in a wide variety of flowering plant species and environments, and to develop new perspectives on its ecological, evolutionary and functional role in plant populations. Recent studies suggest flower herbivory needs to be conceptualized as different from leaf herbivory [1], as they represent ecological interactions that differ in important ways. For example, unlike leaf herbivores that indirectly affect plant reproduction through alteration of the photosynthetic capacity and water balance function [2,3], floral herbivores influence not only plant physiology but link most processes related with plant reproduction through damaging primary reproductive tissues such as pistils, anthers and ovules [4–6]. Likewise, by consuming accessory tissues such as petals, sepals, or bracts, flower herbivores change flower display and floral integration, which often discourage pollinators to visit damaged flowers and reduce substantially plant reproduction [1,7–11]. Even though flower herbivory can decrease plant fitness to degrees comparable with or exceeding leaf herbivory [12–16], relatively few studies have examined its importance for processes that occur beyond the scale of local populations [17], such as those involving colonization of new habitats and establishment in novel environments. This omission is unfortunate as the fate of invasive species in new habitats is determined, at least in part, by the biotic scenario and the balance between mutualistic and antagonistic interactions found in novel communities [18–20].

One of the most important frameworks linking antagonistic interactions and invasion processes is the enemy-release hypothesis (ERH hereafter). This hypothesis indicates that exotic plants may suffer less herbivory and fitness costs in their novel ranges compared to co-occurring native plants, because invaders leave behind natural enemies present in their original range [18]. In the absence of natural enemies, the hypothesis predicts that plants in novel habitats may benefit from reduced herbivore regulation, leading to increased densities that may result in population spread. While a large number of studies have been carried out to test this hypothesis [19–24], conclusions have provided mixed results [25,26], suggesting that the ERH may not be applicable to all cases. For instance, it has been reported that the ERH is a context-dependent hypothesis, where studies of herbivory at the local community level (i.e., comparing native and introduced species co-occurring in a community) rarely support the hypothesis in comparison to tests performed at a larger biogeographical scale (i.e., comparing the same plant species in its natural and introduced range) [27]. Likewise, results from a meta-analysis found that native species have better performance than invasive alien ones, suggesting that native species are more tolerant to damage [28]. To our knowledge, the only study addressing the ERH in the context of flower herbivory is that of Sowell and Wolfe (2010) on four *Ipomoea* species at the community level. Their main finding indicates that the intensity of floral herbivory was contingent upon the residence status of the plant species. The native *Ipomoea* species experienced higher florivory intensity and had a stronger reproductive impact than non-native species. In principle, this result would suggest that the ERH, first developed in the context of foliar herbivory, might also apply to studies of flower herbivory, as proposed by McCall and Irwin (2006). However, it is likely, at least in principle, that generalist flower herbivores found in novel habitats shift onto newly introduced plants, causing a stronger fitness cost than the observed original habitat. Unfortunately, no attempt has been made to quantitatively synthesize the existing evidence for floral herbivory at broad spatial scales, and in consequence, no generalization is possible regarding the specific effect of florivory for invasion processes.

In this study we present the results of a meta-analysis on the fitness impact of flower herbivory on native and exotic plant populations. While our primary emphasis is on the role of the provenance of plant species, we also examine the importance of additional moderators such as the type of damage inflicted to flowers and the ecological interaction responsible for flower damage. More specifically, in this study we will examine the magnitude and direction of overall florivory effects across studies, and will address the extent to which such effects depend on the methodology used in studies of flower herbivory (natural or simulated herbivory), the plant response to the type of flower herbivory (florivory or nectar robbing), and the plant residence status (native and exotic).

## Materials and Methods

We searched the electronic databases ISI Web of Science (1981- August 2013) and Scopus (1960- August 2013) for the following keywords: “flower herbivory”, “floral herbivory”, “florivory”, “petal herbivory” and “nectar rob*”. In addition, we examined the reference list of narrative reviews [1,14,29,30]. To be included in the meta-analysis, the published study had to fulfill the following four criteria: 1) to describe the effect of the type of floral herbivory (florivory or nectar robbing) on plant fitness (e.g., seed set, fruit set, seed production per plant, fruit production per plant, pollen deposition on stigma, pollen removal and export); 2) to have at least two treatments, namely, control (undamaged flowers) and florivory or nectar robbing (natural or experimental flower damage); 3) to report the mean, sample size, and dispersion measure (standard deviation or standard error) of each treatment or the statistics of the test employed indicating the direction of the effect and its significance level. When information was presented in graphs only, we used Graph Click version 3.0 (available at: http://www.arizonasoftware.ch/graphclick/download.html) to extract the mean and dispersion measures, and 4) to present herbivory not performed by ungulates as they often browse and damage plants in a broader scale than flower units, which is the focus of this study. After inspection of 214 papers, we found 51 that satisfied the four criteria indicated above, gathering 83 records from them. We included more than one record per study only in cases where different plant species and/or populations were studied in the same research and when the study included female and male fitness estimations. When the same population was measured in different years, we computed a mean effect size across years to be included in the general analysis (see Table 1 for details). In addition to the 83 case studies extracted from the literature, we included 60 records corresponding to unpublished data (S1 Table). In total, we gathered 143 records from 41 families, 78 genera and 96 plant species.

**Table 1:** Major characteristics of studies included in the meta-analysis. Asterisk indicates mean values across years for the same study, species, site and response variable.

Ref #	Peer reviewed	Authors	Plant species	Family	Residence status	Type of damage	Response variable	Hedges'd	Variance	Total sample size
[4]	yes	Krupnick & Weis 1999	*Isomeris arborea*	Capparaceae	Native	Florivory	Pollen grains per stamen	-0.531	0.030	139
[5]	yes	Maron et al 2002	*Cirsium occidentale*	Asteraceae	Native	Florivory	Viable seeds (old dune)	-1.881	0.115	50
[5]	yes	Maron et al 2002	*Cirsium occidentale*	Asteraceae	Native	Florivory	Viable seeds (new dune)	-0.732	0.089	48
[15]	yes	Mothershead & Marquis 2000	*Oenothera macrocarpa*	Onagraceae	Native	Florivory	Fruit set	-0.445	0.025	514
[16]*	yes	Hendrix & Trapp 1989	*Pastinaca sativa*	Apiaceae	Exotic	Florivory	Recruitment	-0.651	0.164	20
[16]	yes	Hendrix & Trapp 1989	*Pastinaca sativa*	Apiaceae	Exotic	Florivory	Pollen grains per stamen	0.323	0.029	141
[53]	yes	Caballero et al 2013	*Tristerix aphyllus*	Loranthaceae	Native	Nectar robbery	Fruit set	0.093	0.127	32
[54]	yes	Hendrix 1984	*Heracleum lanatum*	Apiaceae	Native	Florivory	Seeds per plant	-0.331	0.152	27
[55]	yes	Ashman et al 2004	*Fragaria virginiana*	Rosaceae	Native	Florivory	Fruit Number	-0.161	0.053	76
[56]	yes	Hendrix & Trapp 1981	*Pastinaca sativa*	Apiaceae	Exotic	Florivory	Seed production	2.154	0.578	11
[57]	yes	Krupnick & Weis 1998	*Isomeris arborea*	Capparaceae	Native	Florivory	Viable seeds per fruit	0.432	0.117	35
[58]*	yes	Louda & Potvin 1995	*Cirsium canescens*	Asteraceae	Native	Florivory	Viable undamaged seeds	-0.819	0.048	81
[59]	yes	Burkle et al 2007	*Delphinium nuttallianum*	Ranunculaceae	Native	Nectar robbery	Seeds per fruit	-0.396	0.138	38
[59]	yes	Burkle et al 2007	*Linaria vulgaris*	Scrophulariaceae	Exotic	Nectar robbery	Seeds per fruit	1.538	0.259	20
[60]	yes	Deng et al 2004	*Alpinia kwangsiensis*	Zingiberaceae	Native	Nectar robbery	Fruit set	-1.930	0.977	6
[61]	yes	Maloof 2001	*Corydalis caseana*	Fumariaceae	Native	Nectar robbery	Seeds per fruit	-0.688	0.265	16
[62]	yes	Navarro 2001	*Macleania bullata*	Ericaceae	Native	Nectar robbery	Fruit set	-2.068	0.018	344
[63]	yes	Richardson 2004	*Chilopsis linearis*	Bignoniaceae	Native	Nectar robbery	Pollen tubes per style	0.420	0.069	64
[64]	yes	Traveset et al 1998	*Fuchsia magellanica*	Onagraceae	Native	Nectar robbery	Fruit set	-1.947	0.295	20
[65]	yes	Zhang et al 2009	*Corydalis tomentella*	Fumariaceae	Native	Nectar robbery	Seed set	-0.257	0.022	191
[65]	yes	Zhang et al 2009	*Corydalis incisa*	Fumariaceae	Native	Nectar robbery	Seed set	0.046	0.017	234
[65]	yes	Zhang et al 2009	*Corydalis ternatifolia*	Fumariaceae	Native	Nectar robbery	Seed set	-0.197	0.017	236
[66]	yes	Amsberry & Maron 2006	*Balsamorhiza sagittata*	Asteraceae	Native	Florivory	Seeds per plant (site 1)	-0.197	0.033	120
[66]	yes	Amsberry & Maron 2006	*Balsamorhiza sagittata*	Asteraceae	Native	Florivory	Seeds per plant (site 2)	-0.471	0.034	120
[66]	yes	Amsberry & Maron 2006	*Balsamorhiza sagittata*	Asteraceae	Native	Florivory	Seeds per plant (site 3)	-0.358	0.034	120
[66]	yes	Amsberry & Maron 2006	*Balsamorhiza sagittata*	Asteraceae	Native	Florivory	Seeds per plant (site 4)	0.109	0.033	120
[67]	yes	Valdivia & Niemeyer 2005	*Alstroemeria umbellata*	Alstroemeriaceae	Native	Florivory	Seed set	-0.438	0.011	385
[68]	yes	Fritz & Morse 1981	*Asclepias syriaca*	Asclepiadaceae	Native	Nectar robbery	Pollinia insertions	-0.084	0.148	27
[69]	yes	Navarro 2000	*Anthyllis vulneraria*	Fabacecae	Native	Nectar robbery	Fruit set	1.214	0.070	68
[70]	yes	Temeles & Pan 2002	*Impatiens capensis*	Balsaminaceae	Native	Nectar robbery	Pollen on stigmas	-0.031	0.051	79
[71]	yes	Utelli & Roy 2001	*Aconitum lycoctonum*	Ranunculaceae	Native	Nectar robbery	Seeds per fruit	-0.138	0.074	54
[72]	yes	Zhang et al 2007	*Glechoma longituba*	Lamiaceae	Native	Nectar robbery	Pollen in anthers	0.112	0.050	80
[72]	yes	Zhang et al 2007	*Glechoma longituba*	Lamiaceae	Native	Nectar robbery	Fruit set	-0.227	0.003	1159
[72]	yes	Zhang et al 2007	*Glechoma longituba*	Lamiaceae	Native	Nectar robbery	Seed set	-0.632	0.420	10
[73]	yes	de Waal et al 2012	*Babiana ringens*	Iridaceae	Native	Florivory	Seed set	0.096	0.067	60
[74]	yes	Navarro et al 1993	*Petrocoptis grandiflora*	Caryophyllaceae	Native	Nectar robbery	Fruit set	1.673	0.100	54
[75]	yes	Wise et al 2008	Solanum carolinense	Solanaceae	Native	Florivory	Fruits per plant	-1.596	0.110	48
-	no	Navarro, L. unpublished data	*Centropogon granulosus*	Campanulaceae	Native	Nectar robbery	Fruit set	-2.433	0.696	10
-	no	Navarro, L. unpublished data	*Barleria cristata*	Acanthaceae	Exotic	Nectar robbery	Fruit set	-1.989	0.272	22
-	no	Navarro, L. unpublished data	*Asystasia gangetica*	Acanthaceae	Exotic	Nectar robbery	Fruit set	-1.407	0.499	10
-	no	Navarro, L. unpublished data	*Alloplectus tetragonoides*	Gesneriaceae	Native	Nectar robbery	Fruit set	-0.051	0.400	10
-	no	Navarro, L. unpublished data	*Aloe secundiflora*	Xanthorrhoeaceae	Native	Nectar robbery	Fruit set	0.708	0.213	20
-	no	Navarro, L. unpublished data	*Aloe vera*	Xanthorrhoeaceae	Exotic	Nectar robbery	Fruit set	-1.036	0.351	13
-	no	Navarro, L. unpublished data	*Alpinia purpurata*	Zingiberaceae	Exotic	Nectar robbery	Fruit set	-2.902	0.684	12
-	no	Navarro, L. unpublished data	*Alpinia purpurata*	Zingiberaceae	Exotic	Nectar robbery	Fruit set	-3.276	1.204	8
-	no	Navarro, L. unpublished data	*Anthirrinun majus*	Plantaginaceae	Native	Nectar robbery	Fruit set	-0.516	0.258	16
-	no	Navarro, L. unpublished data	*Aquilegia vulgaris*	Ranunculaceae	Native	Nectar robbery	Fruit set	-2.307	0.196	34
-	no	Navarro, L. unpublished data	*Capanea grandiflora affinis*	Gesneriaceae	Native	Nectar robbery	Fruit set	-1.032	0.378	12
-	no	Navarro, L. unpublished data	*Castilleja angustifolia*	Orobanchaceae	Native	Nectar robbery	Fruit set	-2.115	0.329	19
-	no	Navarro, L. unpublished data	*Castilleja sp2*	Orobanchaceae	Native	Nectar robbery	Fruit set	-1.849	0.571	10
-	no	Navarro, L. unpublished data	*Cavendishia grandifolia*	Ericaceae	Native	Nectar robbery	Fruit set	-0.719	0.304	14
-	no	Navarro, L. unpublished data	*Ceratostema fasciculatum*	Ericaceae	Native	Nectar robbery	Fruit set	-2.062	0.613	10
-	no	Navarro, L. unpublished data	*Columnea glabra*	Gesneriaceae	Native	Nectar robbery	Fruit set	-2.979	0.796	11
-	no	Navarro, L. unpublished data	*Columnea minor*	Gesneriaceae	Native	Nectar robbery	Fruit set	-2.549	0.604	12
-	no	Navarro, L. unpublished data	*Delphinium halteratum*	Ranunculaceae	Native	Nectar robbery	Fruit set	-1.422	0.147	34
-	no	Navarro, L. unpublished data	*Disterigma stereophylla*	Ericaceae	Native	Nectar robbery	Fruit set	-0.288	0.227	18
-	no	Navarro, L. unpublished data	*Drymonia coriacea*	Gesneriaceae	Native	Nectar robbery	Fruit set	-2.257	0.468	14
-	no	Navarro, L. unpublished data	*Escallonia rubra*	Escalloniaceae	Exotic	Nectar robbery	Fruit set	-5.984	1.564	14
-	no	Navarro, L. unpublished data	*Hamelia patens*	Rubiaceae	Native	Nectar robbery	Fruit set	-0.550	0.380	11
-	no	Navarro, L. unpublished data	*Jasminum fruticans*	Oleaceae	Native	Nectar robbery	Fruit set	-1.436	0.419	12
-	no	Navarro, L. unpublished data	*Justicia aurea*	Acanthaceae	Native	Nectar robbery	Fruit set	-0.589	0.279	15
-	no	Navarro, L. unpublished data	*Justicia pectoralis*	Acanthaceae	Native	Nectar robbery	Fruit set	-0.354	0.290	14
-	no	Navarro, L. unpublished data	*Kalanchoe pinnata*	Crassulaceae	Exotic	Nectar robbery	Fruit set	-0.917	0.030	187
-	no	Navarro, L. unpublished data	*Kalanchoe pinnata*	Crassulaceae	Exotic	Nectar robbery	Fruit set	-2.841	0.449	18
-	no	Navarro, L. unpublished data	*Kalanchoe pinnata*	Crassulaceae	Exotic	Nectar robbery	Fruit set	-0.886	0.139	33
-	no	Navarro, L. unpublished data	*Kniphofia thomsonii*	Xanthorrhoeaceae	Exotic	Nectar robbery	Fruit set	-0.740	0.480	9
-	no	Navarro, L. unpublished data	*Lamiun maculatum*	Lamiaceae	Native	Nectar robbery	Fruit set	-1.248	0.154	31
-	no	Navarro, L. unpublished data	*Lantana camara*	Verbenaceae	Exotic	Nectar robbery	Fruit set	-2.838	0.446	18
-	no	Navarro, L. unpublished data	*Lantana camara*	Verbenaceae	Exotic	Nectar robbery	Fruit set	-4.188	0.912	14
-	no	Navarro, L. unpublished data	*Linaria triornitophora*	Scrophulariaceae	Native	Nectar robbery	Fruit set	-0.151	0.251	16
-	no	Navarro, L. unpublished data	*Linaria vulgaris*	Scrophulariaceae	Native	Nectar robbery	Fruit set	-1.069	0.290	16
-	no	Navarro, L. unpublished data	*Lithodora prostrata*	Boraginaceae	Native	Nectar robbery	Fruit set	-0.988	0.077	59
-	no	Navarro, L. unpublished data	*Lonicera periclymenum*	Caprifoliaceae	Native	Nectar robbery	Fruit set	-0.284	0.094	43
-	no	Navarro, L. unpublished data	*Macleania stricta*	Ericaceae	Native	Nectar robbery	Fruit set	-2.276	0.275	24
-	no	Navarro, L. unpublished data	*Melampyrum nemorosum*	Orobanchaceae	Native	Nectar robbery	Fruit set	-0.242	0.270	15
-	no	Navarro, L. unpublished data	*Melampyrum polonicum*	Orobanchaceae	Native	Nectar robbery	Fruit set	-0.124	0.223	18
-	no	Navarro, L. unpublished data	*Melampyrum pratense*	Orobanchaceae	Native	Nectar robbery	Fruit set	-0.738	0.225	19
-	no	Arroyo, J. unpublished data	*Narcissus papyraceus*	Amaryllidaceae	Native	Nectar robbery	Fruit set	-1.344	0.111	44
-	no	Navarro, L. unpublished data	*Nicotiana glauca*	Solanaceae	Exotic	Nectar robbery	Fruit set	-1.559	0.435	12
-	no	Navarro, L. unpublished data	*Odontonema strictum*	Acanthaceae	Native	Nectar robbery	Fruit set	-2.526	0.402	18
-	no	Navarro, L. unpublished data	*Palicourea croceoides*	Rubiaceae	Native	Nectar robbery	Fruit set	-0.451	0.205	20
-	no	Navarro, L. unpublished data	*Passiflora mixta*	Passifloraceae	Native	Nectar robbery	Fruit set	-0.762	0.482	9
-	no	Navarro, L. unpublished data	*Pedicularis sylvatica*	Scrophulariaceae	Native	Nectar robbery	Fruit set	-1.359	0.164	30
-	no	Navarro, L. unpublished data	*Ruellia tuberosa*	Acanthaceae	Native	Nectar robbery	Fruit set	-0.244	0.270	15
-	no	Navarro, L. unpublished data	*Russelia equisetiformis*	Scrophulariaceae	Exotic	Nectar robbery	Fruit set	-0.205	0.201	20
-	no	Navarro, L. unpublished data	*Salvia haenkei*	Lamiaceae	Native	Nectar robbery	Fruit set	-0.713	0.425	10
-	no	Navarro, L. unpublished data	*Salvia verbenaca*	Lamiaceae	Native	Nectar robbery	Fruit set	-0.817	0.207	21
-	no	Navarro, L. unpublished data	*Siphocampylus aureus*	Campanulaceae	Native	Nectar robbery	Fruit set	-0.432	0.274	15
-	no	Navarro, L. unpublished data	*Siphocampylus aureus*	Campanulaceae	Native	Nectar robbery	Fruit set	-0.339	0.422	10
-	no	Navarro, L. unpublished data	*Sphyrospermun sp*.	Ericaceae	Native	Nectar robbery	Fruit set	-1.382	0.413	12
-	no	Navarro, L. unpublished data	*Stachytarpheta jamaicensis*	Verbenaceae	Native	Nectar robbery	Fruit set	-0.269	0.150	27
-	no	Navarro, L. unpublished data	*Thunbergia grandiflora*	Acanthaceae	Exotic	Nectar robbery	Fruit set	-0.132	0.401	10
-	no	Navarro, L. unpublished data	*Thunbergia grandiflora*	Acanthaceae	Exotic	Nectar robbery	Fruit set	-0.617	0.349	12
-	no	Navarro, L. unpublished data	*Trifolium campestre*	Fabacecae	Native	Nectar robbery	Fruit set	0.199	0.096	42
-	no	Navarro, L. unpublished data	*Weigela florida*	Caprifoliaceae	Exotic	Nectar robbery	Fruit set	-5.483	0.634	30
-	no	Navarro, L. unpublished data	*Wisteria sinensis*	Fabacecae	Exotic	Nectar robbery	Fruit set	-8.033	1.648	22
-	no	Navarro, L. unpublished data	*Duranta erecta*	Verbenaceae	Exotic	Nectar robbery	Fruit set	-2.334	0.306	22

* Values correspond to mean across years for the same study, species, site, and response variable

We calculated the Hedges unbiased standardized mean difference effect size for each data set to estimate the difference in the mean fitness of undamaged and damaged plants [31]. The effect size d was expressed as follows:

$$d=\frac{{\bar{X}}_{1}-{\bar{X}}_{2}}{S_{pooled}}\;J$$

in which ${\bar{X}}_{1}$ and ${\bar{X}}_{2}$ are the sample means of the two groups (damaged and undamaged plants, respectively) and S_pooled_ their pooled standard deviation, expressed as:

$$S_{pooled}=\sqrt{\frac{s_{1}^{2}(n_{1}-1)+s_{2}^{2}(n_{2}-1)}{n_{1}n_{2}-2}}$$

where n_1_ and n_2_ are the sample sizes and s_1_ and s_2_ are the standard deviations of the two groups corrected for sample size with the correction factor j [32]. The weighting factor J was calculated as:

$$J=1-\frac{3}{4(n_{1}n_{2}-2)-1}$$

In this study, a positive effect size indicates that plant fitness is lower in control plants (i.e., flowers not damaged) compared to treatment (damaged) plants, while a negative effect size implies a fitness cost for the damage plants as compared to the control.

We first performed a general analysis to describe the global effect of florivory on plant fitness, and then incorporated moderators. We evaluated the effect of three categorical variables, namely: 1) Design, including two levels: natural herbivory damage and simulated damage (clipping, floral and petal removal and simulated nectar robbing). The aim of this categorization was to determine whether artificial damage faithfully mimics the natural flower damage experienced by plants [33]; 2) Residence status, with two levels: native (a species that inhabits its natural range) and exotic (a species that has been introduced to novel habitats outside its natural range). The aim of this categorization was to assess whether the fitness impact of floral damage was contingent on the provenance of the plant species to the place where the study was performed. When residence status was not informed in the article, we looked for information about the native distribution of the species involved in other publications or data bases; 3) Type of damage, using two levels: florivory (damage to petals, sepals or any other floral attraction trait) and nectar robbery (damage at the corolla base to access the nectar chamber). Potential bias in the representation of cases among moderator levels was evaluated in a Fisher’s exact test (S2 Fig). To examine whether variation in effect size was attributable to differences between moderator levels, we calculated between-group homogeneity (Q_B_) and tested it against the χ^2^ distribution with N (the number of levels) minus one degrees of freedom [34].

As most experimental reports on flower herbivory have been performed on a per species basis in one locality, often omitting information related to the community context, our meta-analysis was restricted to native and exotic plants that do not necessarily co-occur in local communities. Therefore we considered the effects of flower herbivory on plant species that could be clearly classified as native or exotic to the region where studies were conducted, regardless of community co-occurrence. We used a mixed-effect model for the analysis of moderators, assuming a random effect within moderator levels because measurements were recorded from a variety of plant species and environments, and a fixed effect to compare moderator levels based on the idea that we gathered all possible categories into two levels for each moderator rather than a random sample of the possible existing levels [35,36]. Additionally, in order to evaluate if our results were real or resulted from non-independent phylogeny effects, we performed a second analysis where “family” was incorporated as a random factor in the model. This analysis was carried out using the package metafor in R [37]. Publication bias was estimated using the Pearson’s correlation coefficient and the funnel plot method, which indicates that in the absence of bias, effect size should not correlate with sample size [38]. When the mean effect size significantly differed from zero we calculated Rosenthal’s fail-safe number, which represents the number of unpublished studies with zero effect needed to reverse the significant effect revealed in the meta-analysis [39]. When the fail-safe number was greater than 5n + 10 (where n is the number of records in the analysis), it may be concluded that the results were robust against publication bias [40]. All analyses were performed in Comprehensive Meta-Analysis v. 2.0 (Biostat Inc.)

## Results

**Fig 1:**
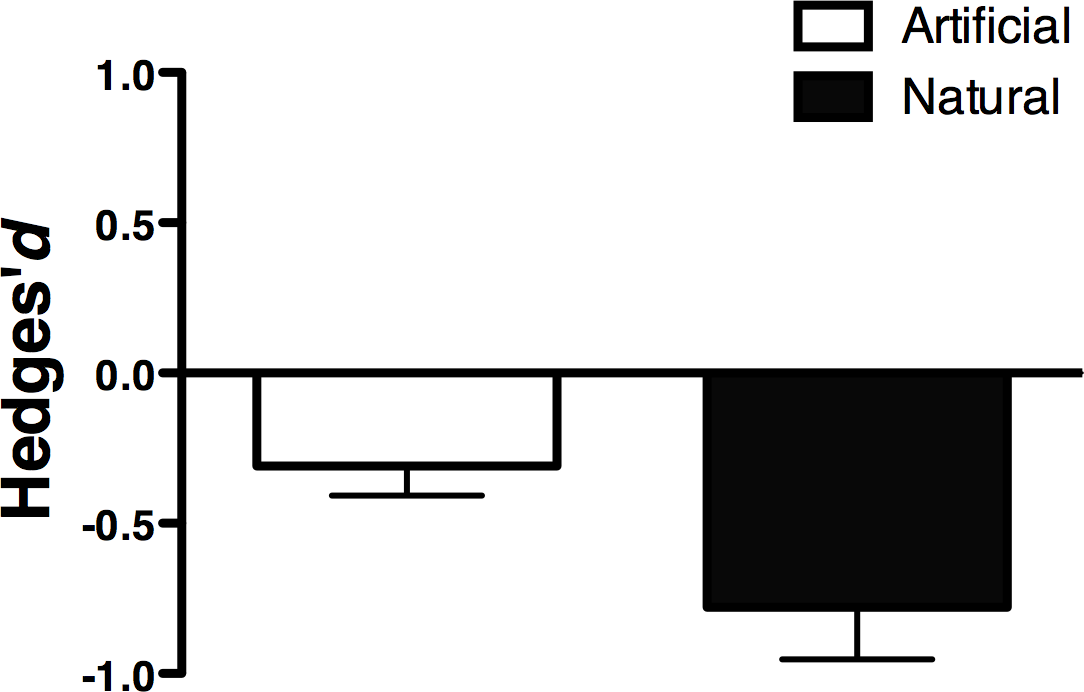
Mean effect size of flower herbivory on plant fitness in studies using natural and artificial herbivory. Bars around means indicate 95% confidence intervals. Numbers indicate the number of records.

We performed a general analysis including the 143 records to evaluate if natural and artificial damage had different effects on plant reproductive success. Floral damage had a significant cost on plant reproductive success (d = -0.535, N = 143, p < 0.001; heterogeneity, Q = 1033.9, df = 142, p<0.001). Additionally, our results indicated that the impact of natural floral herbivory on plant fitness was greater than the artificial damage (Fig 1, Natural: d = -0.780, N = 97, p < 0.001; Artificial: d = -0.309, N = 46, p < 0.001) and this difference was statistically significant (Q_B_ = 22.1, df = 1, p < 0.001), indicating that natural and artificial damage differ in the magnitude of effects. Therefore, only data from studies evaluating natural damage were included in subsequent analyses. The original dataset was reduced by 32% and included 97 reports from 29 publications, corresponding to 81 different plant species from 66 genera and 35 families (Table 1).

**Fig 2:**
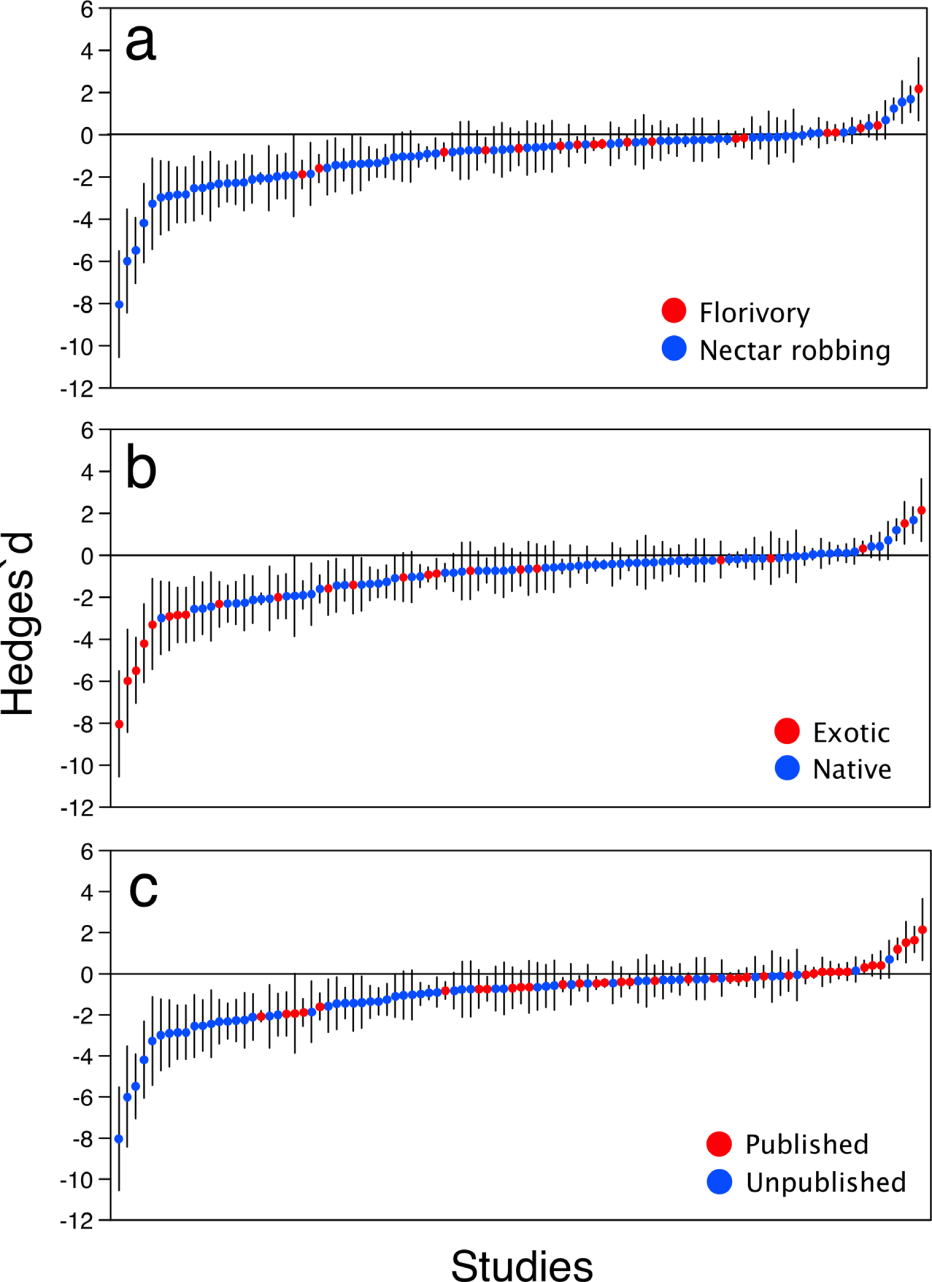
Distribution of Hedges’d effects of flower herbivory on plant fitness arranged in increasing order for (a) type of damage, (b) origin, and (c) data source. Bars indicate 95% CI. The zero line is presented for reference of statistical significance.

**Fig 3:**
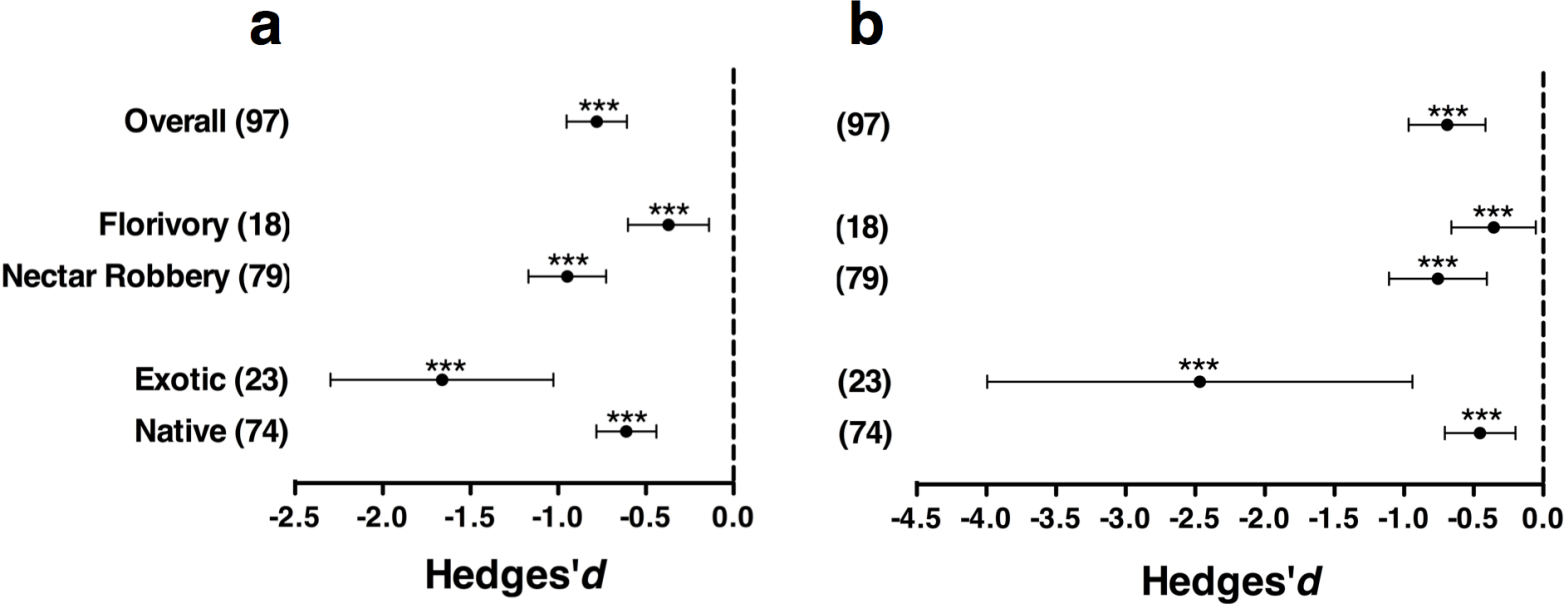
Plots of mean effect sizes for levels of both moderators at the (a) species level (97 reports, 81 species) and (b) species level, but including “family” as a random factor. As some families are represented in the two levels of the moderators, sample size exceeds the overall sample size in the family-level analysis (N = 35). Bars around means denote 95% confidence intervals. Parentheses indicate sample size. *** p < 0.001; ** p < 0.01; * p < 0.05

The distribution of Hedges’ d values revealed a predominance of negative (44.3%) and neutral effects (51.6%), with a minor contribution of positive effects (4.1%) (Fig 2). Among significant effects there was a predominance of negative ones (85.5% of cases). When examined across all studies, the mean effect size was significantly less than zero (Fig 3A). Regarding the type of damage, florivory and nectar robbery imposed a significant cost to plant reproduction (d = -0.37, N = 18, p = 0.002; d = -0.95, N = 79, p < 0.001, respectively) but differed in the magnitude of their effects (Q_B_ = 12.5 df = 1, p < 0.001); the impact of nectar robbing was greater than that of florivory (Fig 3A). The inclusion of the residence status as moderator revealed that floral herbivory had a significant fitness cost on native and exotic plants, and a significant heterogeneity in the magnitude of effects between levels (Q_B_ = 9.8, df = 1, p = 0.002). The mean fitness impact of flower herbivory upon exotics was three-fold stronger than on native plants (d = -1.66, N = 23, p < 0.001 versus d = -0.61, N = 74, p < 0.001, respectively, Fig 3A). Exotic plants had more variable effects than native plants (Bartlett's K-squared = 37.9, df = 1, p < 0.001). When data were analyzed incorporating “Family” as a random factor, the results showed the same tendency as the first analysis (Fig 3B). The presence of natural floral damage strongly reduced plant fitness (d = -0.61, N = 97, p < 0.001) and the separate effects of nectar robbing and florivory upon plant fitness were also negative (d = -0.76, N = 79, p <0.0001; d = -0.36, N = 18, p = 0.02, respectively), and as in the first analysis, nectar robbers and florivores differed in their effects upon plant fitness (Q_B_ = 4.16, df = 1, p = 0.04). Regarding residence status, flower herbivores reduced the fitness of exotic and native plants (exotics: d = -2.47, N = 23, p = 0.002; native: d = -0.45, N = 74, p = 0.0005), and such impact was stronger on exotic than native plants (Q_B_ = 13.3, df = 1, p = 0.0003)

Effect size was not associated with sample size (Pearson’s product-moment correlation coefficient, r = 0.139, N = 97, p = 0.174, indicating absence of publication bias. Visual inspection of the funnel plot suggests potential selection against small-sample studies that demonstrate positive effects of flower herbivory on plant fitness (S1 Fig). Rosenthal’s fail-safe number indicates that 8569 unpublished studies with zero effect would be necessary to reverse the significance of effects. As this number exceeds by far the expected value for absence of publication bias (5 x 97 + 10 = 495), we conclude that our results were robust to publication omission.

## Discussion

The distribution of Hedges’ d values revealed a predominance of negative and neutral effects, which is consistent with previous conclusions from narrative reviews indicating that the effect of flower herbivory may vary from negative to neutral, and only rarely may benefit plant reproductive success [1]. As expected, the mean effect size was negative, corroborating the overall detrimental impact of flower herbivores on plant fitness. When effects were analyzed in the context of natural and artificial flower herbivory, both methodologies imposed an important cost to plant reproductive success, albeit the fitness cost of natural herbivory was two-fold stronger than that of artificial damage, cautioning the longstanding assumption that artificial flower damage can be used as a legitimate surrogate for the natural damage imposed by flower herbivores. This result alerts experimental studies using simulated flower herbivory, such as clipping or petal removal, as they do not completely mimic the plant response to natural flower herbivory and may underestimate the real fitness impact of herbivores (see also [41,42]). It is likely that plant responses following natural flower damage increase the susceptibility to subsequent antagonistic interactions such as foliar herbivory and seed predation [43,44], especially if the new consumers are able to detect chemical signals associated with floral damage such as volatiles released by the corolla tissues. Similarly, assuming the same concepts of resistance and tolerance can be extended to understand how plants and flowers cope with damage by florivores [1], induced defenses that deter florivores may also deter pollinators or simply impose a higher fitness cost related with the production and mobilization of such defenses [1,45]. Such a hypothesis clearly requires experimental investigation.

A previous meta-analysis performed on a broad review of the invasion literature including plants, invertebrates and vertebrate species examined whether exotics really have a low diversity of enemies in the new habitats, as predicted by the ERH [27]. The authors performed tests that compared the enemy species diversity between exotic and native populations of the same species (biogeographical level), and between exotic and native species co-occurring within the same community (community level). Their results supported the enemy release hypothesis at the biogeographical level only, indicating that the phenomenon seems to be contingent on the scale at which studies are performed. In consequence, in spite of the overemphasis received in the literature of invasion, the ERH seems to be insufficient to account for the inherent complexity of the invasion process. In our meta-analysis, the paucity of studies using the same plant species in native and novel habitats as well as the limited number of studies at the community level precluded examination of the importance of the residence status at the resolution levels suggested by Colautti et al. (2004). Notwithstanding, the effects of floral herbivory on plant fitness were clear and significantly modulated by plant origin and stronger on exotic than native species (Fig 3). It is likely that the ample variation in the effect size of exotics results from the limited number of studies in this category (23) in comparison to native species (74). The stronger effects on exotics however, is intriguing and may be explained, at least in part, if exotic plants are more susceptible to enemies in novel habitats and/or herbivores in novel habitats converge to the introduced plant. The evidence is mixed in this regard. On one hand, Cappuccino and Carpenter (2005) analyzed the importance of leaf herbivory on 18 exotic plant species divided into invasive and non-invasive depending on their spread in the novel habitat. Their results indicate that invasive plants suffered 96% less leaf damage than non-invasive exotic species. In the same line, Sugiura (2010) examined the incidence of herbivorous insect species on invasive and native plant species. The results indicated that herbivorous insects were mainly associated with native and indigenous species, hence confirming a critical element of the ERH. On the other hand, recent reviews indicate that exotics are not necessarily devoid of enemies in new habitats, which translate into similar levels of herbivory in coexisting invasive and native plants [28,46]. This effect has been attributed to the high susceptibility of exotic plants to new enemies and to the presence of enemies already present in their original habitat. Under this situation, previous types and levels of defense evolved in original habitats may be less efficient against new natural enemies after arrival [47–49], especially if generalist herbivores shift onto newly introduced plants. The mechanism involved in the greater susceptibility of exotics has been named the “increased susceptibility hypothesis” by Colautti et al. (2004) to denote the effect of invasion bottlenecks that reduce the genetic diversity of polymorphic defenses of exotics, leading to increased susceptibility to the native and introduced enemies found in new habitats. Under such circumstances novel instances of attack may impose high fitness costs on exotic plants in comparison to the more genetically diverse native species. The extent to which a similar situation occurs in studies of flower herbivory needs to be examined in future studies.

Regarding the damage inflicted to flowers, the fitness impact of flower herbivory was significantly modulated by the interaction involved in the herbivory process. Even though both florivores and nectar robbers had significant negative impacts on plant fitness (Fig 3A), nectar robbers had a stronger negative effect on plant fitness than flower consumers. This result is surprising, as unlike florivores that often suppress completely flower reproduction, nectar robbers do not damage reproductive organs but usually restrict their damage to tissues that encompass the nectar reward concealed at the base of floral tubes. There is, however, an important methodological consideration that needs to be taken into account. The effects of florivory and nectar robbing on plant reproduction considered in this meta-analysis were mostly compiled from studies that analyzed florivory and nectar robbing separately but not together. The only study examining potential interaction effects concluded that the exclusion of nectar robbing ants increased the activity of herbivorous beetles on flowers, leading to decreased female fitness [50]. In this way the impact of herbivorous beetles seemed to be contingent on the presence or absence of ants, illustrating the way non-additive effects determine the final outcome of flower herbivory through complex pathways of fitness impact. This is consistent with the increasing experimental evidence indicating that plant-animal interactions often impact plant fitness in non-additive ways, suggesting greater community complexity than previously thought [11,14,51,52] (but see [44,53]). While our meta-analysis revealed broad negative effects of florivory and nectar robbing on plant fitness, the extent to which such effects are canceled when interactions are examined in combination needs to be examined in future studies.

In conclusion, our results revealed that floral herbivores impose a significant cost to plant fitness, which is significantly modulated by the type of damage and plant origin. More specifically, flower herbivores had a higher fitness impact on exotic than on native species, which is not consistent with predictions of the enemy release hypothesis. In consequence, our conclusions point out the limited utility of the ERH to account for the complexity of the invasion process in species subject to flower herbivory. Our results suggest that floral herbivores may play an important but largely unrecognized role in preventing the spread of introduced species in newly colonized areas. More experimental studies evaluating the fitness impact of flower herbivores at biogeographic and community levels are badly needed to extract useful generalizations on the importance of flower herbivory for the invasion process.

## Supporting Information

S1 Fig Funnel plot of sample size (control + treatment) and effect size (Hedges’ d) of flower herbivory on plant fitness. Each dot corresponds to a report. The horizontal line indicates the mean effect size of the global analysis.
(JPG)

S2 Fig Path diagram showing the number of cases on each category. (JPG)

S1 Table Additional information for unpublished data. (DOCX)
