# Supplementary figures and images for: Does Plant Origin Influence the Fitness Impact of Flower Damage? A Meta-Analysis
Source: PLoS One. 2016 Jan 19;11(1):e0146437. doi: 10.1371/journal.pone.0146437 (PMC4718695; doi:10.1371/journal.pone.0146437)

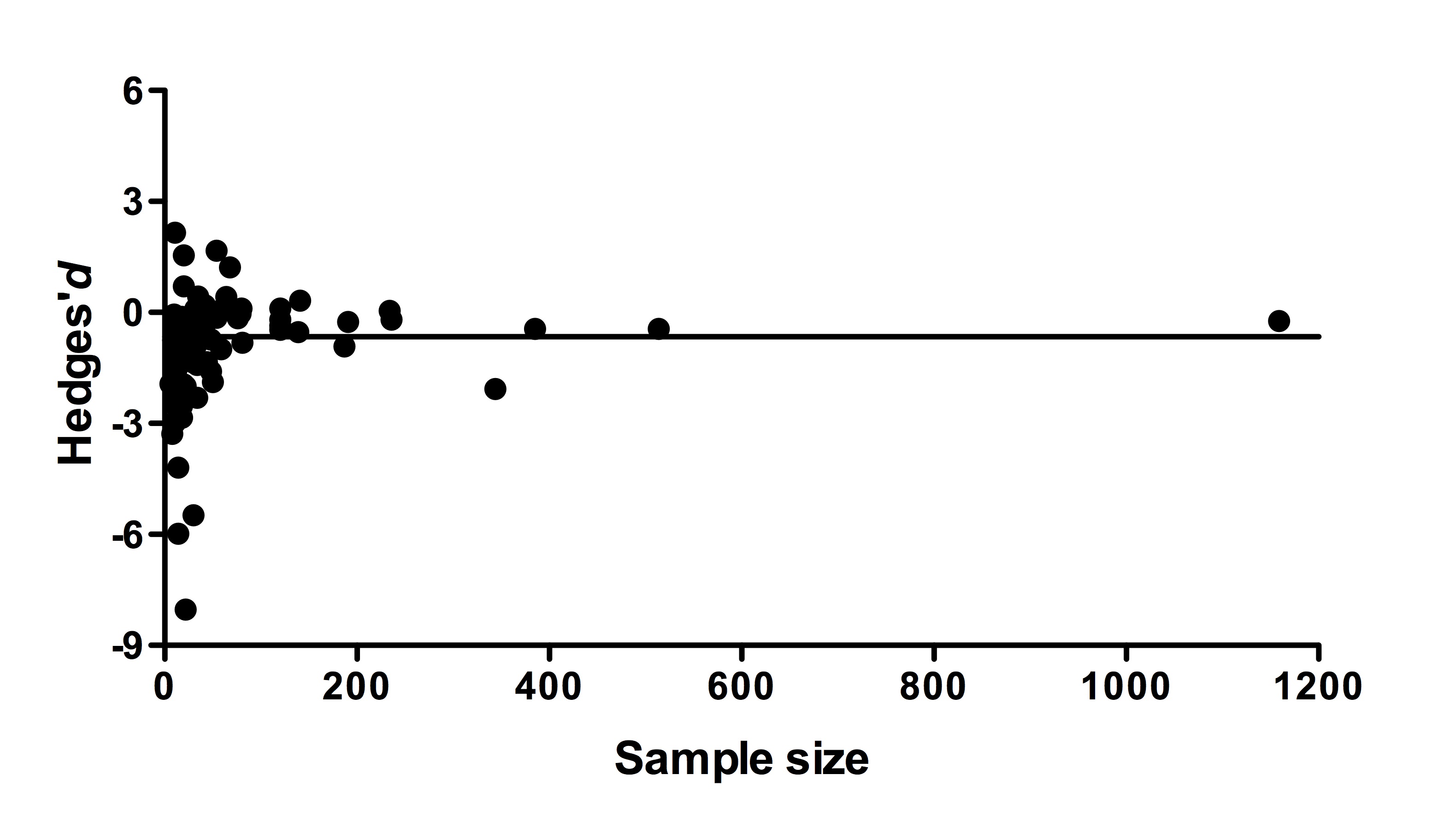

Supplement: S1 Fig — Each dot corresponds to a report. The horizontal line indicates the mean effect size of the global analysis. (JPG) [file pone.0146437.s001.jpg]

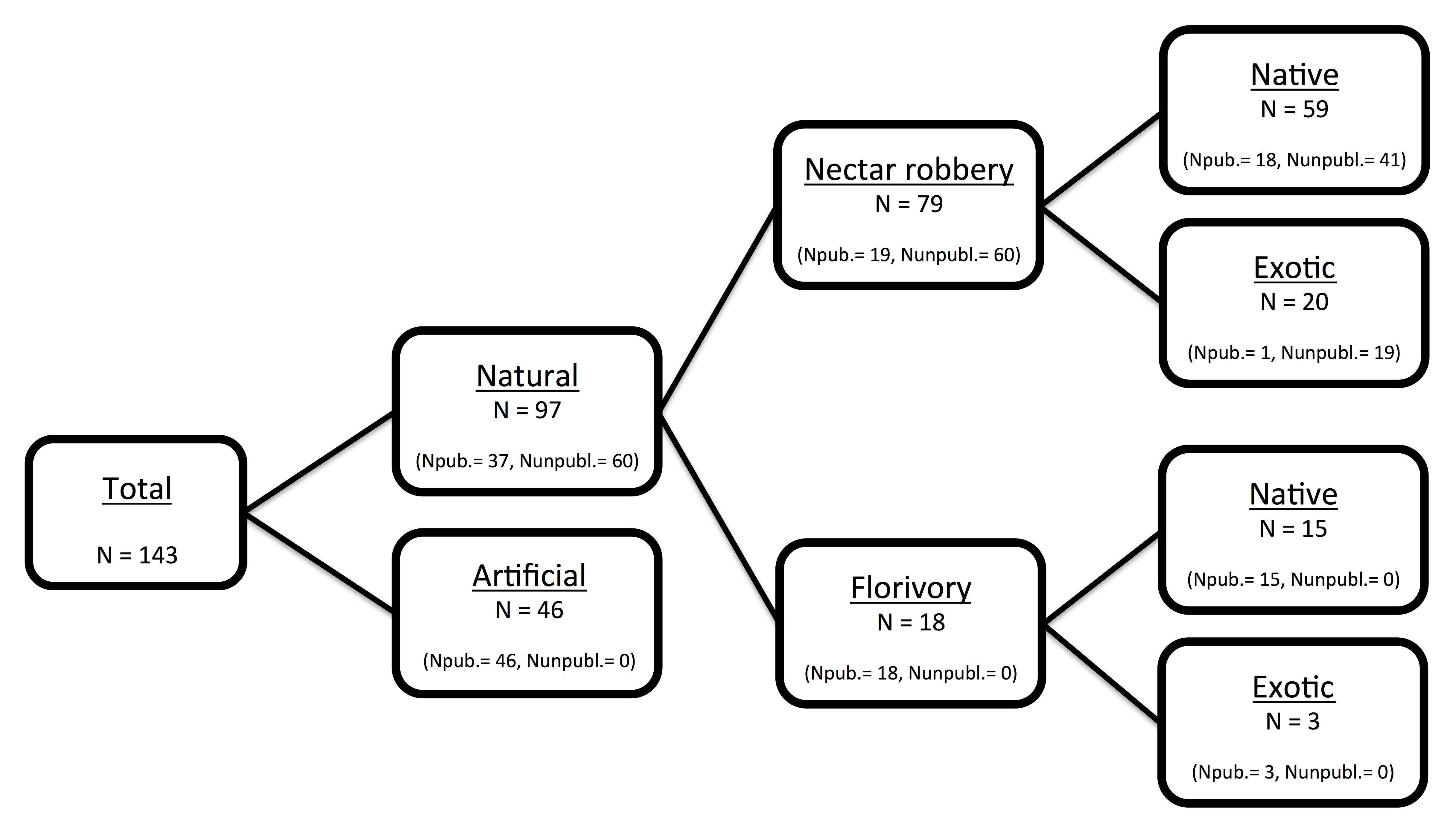

Supplement: S2 Fig — (JPG) [file pone.0146437.s002.jpg]
