# Supplementary material for: Does Plant Origin Influence the Fitness Impact of Flower Damage? A Meta-Analysis
Source: PLoS One. 2016 Jan 19;11(1):e0146437. doi: 10.1371/journal.pone.0146437 (PMC4718695; doi:10.1371/journal.pone.0146437)
Supplement: S1 Table — (DOCX) [file pone.0146437.s003.docx]

| **Table S1** |  | |  | |  | |  | |  | |  | |  | |  | |  | |
| --- | --- | --- | --- | --- | --- | --- | --- | --- | --- | --- | --- | --- | --- | --- | --- | --- | --- | --- |
| **Authors** | | **Plant species** | | **Family** | | **Response variable** | | **Geographical**  **coordinates** | | **Mean ± SD (n)**  **Control group** | | **Mean ± SD (n)**  **Treatment group** | | **Hedges'd** | **Variance** | **Total sample size** | |  |
| Navarro, L. unpublished data | | *Centropogon granulosus* | | Campanulaceae | | Fruit set | | 10°25'N // 84° 1'W | | 0.637 ± 0.208 (5) | | 0.151 ± 0.147 (5) | | -2.433 | 0.696 | 10 | |  |
| Navarro, L. unpublished data | | *Barleria cristata* | | Acanthaceae | | Fruit set | | 18°20'N // 65°43'W | | 0.724 ± 0.153 (11) | | 0.301 ± 0.246 (11) | | -1.989 | 0.272 | 22 | |  |
| Navarro, L. unpublished data | | *Asystasia gangetica* | | Acanthaceae | | Fruit set | | 18°24'N // 66° 2'W | | 0.637 ± 0.093 (5) | | 0.299 ± 0.292 (5) | | -1.407 | 0.499 | 10 | |  |
| Navarro, L. unpublished data | | *Alloplectus tetragonoides* | | Gesneriaceae | | Fruit set | | 1°13'N // 77°59'W | | 0.344 ± 0.159 (5) | | 0.333 ± 0.210 (5) | | -0.051 | 0.400 | 10 | |  |
| Navarro, L. unpublished data | | *Aloe secundiflora* | | Xanthorrhoeaceae | | Fruit set | | 3°26'S // 36°32'E | | 0.164 ± 0.115 (10) | | 0.230 ± 0.052 (10) | | 0.708 | 0.213 | 20 | |  |
| Navarro, L. unpublished data | | *Aloe vera* | | Xanthorrhoeaceae | | Fruit set | | 28°22'N // 16°52'W | | 0.206 ± 0.063 (7) | | 0.100 ± 0.123 (6) | | -1.036 | 0.351 | 13 | |  |
| Navarro, L. unpublished data | | *Alpinia purpurata* | | Zingiberaceae | | Fruit set | | 1°13'N // 77°59'W | | 0.585 ± 0.112 (6) | | 0.178 ± 0.145 (6) | | -2.902 | 0.684 | 12 | |  |
| Navarro, L. unpublished data | | *Alpinia purpurata* | | Zingiberaceae | | Fruit set | | 18°23'N // 66° 2'W | | 0.244 ± 0.063 (5) | | 0.022 ± 0.050 (3) | | -3.276 | 1.204 | 8 | |  |
| Navarro, L. unpublished data | | *Anthirrinun majus* | | Plantaginaceae | | Fruit set | | 42°10'N // 2°34'E | | 0.477 ± 0.080 (8) | | 0.381 ± 0.234 (8) | | -0.516 | 0.258 | 16 | |  |
| Navarro, L. unpublished data | | *Aquilegia vulgaris* | | Ranunculaceae | | Fruit set | | 43° 5'N // 2° 6'W | | 0.645 ± 0.182 (16) | | 0.177 ± 0.212 (18) | | -2.307 | 0.196 | 34 | |  |
| Navarro, L. unpublished data | | *Capanea grandiflora affinis* | | Gesneriaceae | | Fruit set | | 1°13'N // 77°59'W | | 0.625 ± 0.324 (6) | | 0.330 ± 0.184 (6) | | -1.032 | 0.378 | 12 | |  |
| Navarro, L. unpublished data | | *Castilleja angustifolia* | | Orobanchaceae | | Fruit set | | 37°50'N //109°24'W | | 0.628 ± 0.106 (10) | | 0.434 ± 0.060 (9) | | -2.115 | 0.329 | 19 | |  |
| Navarro, L. unpublished data | | *Castilleja sp2* | | Orobanchaceae | | Fruit set | | 9°58'N // 83°50'W | | 0.551 ± 0.055 (5) | | 0.387 ± 0.099 (5) | | -1.849 | 0.571 | 10 | |  |
| Navarro, L. unpublished data | | *Cavendishia grandifolia* | | Ericaceae | | Fruit set | | 1°13'N // 77°59'W | | 0.265 ± 0.100 (7) | | 0.172 ± 0.139 (7) | | -0.719 | 0.304 | 14 | |  |
| Navarro, L. unpublished data | | *Ceratostema fasciculatum* | | Ericaceae | | Fruit set | | 0°54'S // 78°43'W | | 0.176 ± 0.045 (5) | | 0.042 ± 0.070 (5) | | -2.062 | 0.613 | 10 | |  |
| Navarro, L. unpublished data | | *Columnea glabra* | | Gesneriaceae | | Fruit set | | 10°25'N // 84° 1'W | | 0.540 ± 0.118 (7) | | 0.125 ± 0.144 (4) | | -2.979 | 0.796 | 11 | |  |
| Navarro, L. unpublished data | | *Columnea minor* | | Gesneriaceae | | Fruit set | | 1°13'N // 77°59'W | | 0.489 ± 0.107 (6) | | 0.131 ± 0.149 (6) | | -2.549 | 0.604 | 12 | |  |
| Navarro, L. unpublished data | | *Delphinium halteratum* | | Ranunculaceae | | Fruit set | | 42°38'N // 7° 7'W | | 0.387 ± 0.092 (17) | | 0.219 ± 0.135 (17) | | -1.422 | 0.147 | 34 | |  |
| Navarro, L. unpublished data | | *Disterigma stereophylla* | | Ericaceae | | Fruit set | | 1°13'N // 77°59'W | | 0.283 ± 0.076 (10) | | 0.254 ± 0.115 (8) | | -0.288 | 0.227 | 18 | |  |
| Navarro, L. unpublished data | | *Drymonia coriacea* | | Gesneriaceae | | Fruit set | | 1°13'N // 77°59'W | | 0.704 ± 0.163 (7) | | 0.222 ± 0.231 (7) | | -2.257 | 0.468 | 14 | |  |
| Navarro, L. unpublished data | | *Escallonia rubra* | | Escalloniaceae | | Fruit set | | 42°52'N // 8°38'W | | 0.504 ± 0.091 (7) | | 0.061 ± 0.035 (7) | | -5.984 | 1.564 | 14 | |  |
| Navarro, L. unpublished data | | *Hamelia patens* | | Rubiaceae | | Fruit set | | 18°24'N // 66°42'W | | 0.287 ± 0.088 (6) | | 0.235 ± 0.083 (5) | | -0.550 | 0.380 | 11 | |  |
| Navarro, L. unpublished data | | *Jasminum fruticans* | | Oleaceae | | Fruit set | | 42°29'N // 6°50'W | | 0.333 ± 0.046 (6) | | 0.233 ± 0.079 (6) | | -1.436 | 0.419 | 12 | |  |
| Navarro, L. unpublished data | | *Justicia aurea* | | Acanthaceae | | Fruit set | | 1°13'N // 77°59'W | | 0.546 ± 0.151 (8) | | 0.449 ± 0.160 (7) | | -0.589 | 0.279 | 15 | |  |
| Navarro, L. unpublished data | | *Justicia pectoralis* | | Acanthaceae | | Fruit set | | 18°20'N // 65°43'W | | 0.432 ± 0.058 (7) | | 0.404 ± 0.088 (7) | | -0.354 | 0.290 | 14 | |  |
| Navarro, L. unpublished data | | *Kalanchoe pinnata* | | Crassulaceae | | Fruit set | | 0°53'S // 91° 0'W | | 0.286 ± 0.347 (49) | | 0.090 ± 0.139 (138) | | -0.917 | 0.030 | 187 | |  |
| Navarro, L. unpublished data | | *Kalanchoe pinnata* | | Crassulaceae | | Fruit set | | 21°48' // 84°29'W | | 0.458 ± 0.213 (8) | | 0.028 ± 0.038 (10) | | -2.841 | 0.449 | 18 | |  |
| Navarro, L. unpublished data | | *Kalanchoe pinnata* | | Crassulaceae | | Fruit set | | 17°57'N // 66°51'W | | 0.252 ± 0.250 (13) | | 0.092 ± 0.107 (20) | | -0.886 | 0.139 | 33 | |  |
| Navarro, L. unpublished data | | *Kniphofia thomsonii* | | Xanthorrhoeaceae | | Fruit set | | 42°52'N // 8°38'W | | 0.160 ± 0.136 (5) | | 0.071 ± 0.048 (4) | | -0.740 | 0.480 | 9 | |  |
| Navarro, L. unpublished data | | *Lamiun maculatum* | | Lamiaceae | | Fruit set | | 42°52'N // 8°38'W | | 0.543 ± 0.169 (16) | | 0.293 ± 0.220 (15) | | -1.248 | 0.154 | 31 | |  |
| Navarro, L. unpublished data | | *Lantana camara* | | Verbenaceae | | Fruit set | | 17°57'N // 66°51'W | | 0.249 ± 0.040 (9) | | 0.050 ± 0.086 (9) | | -2.838 | 0.446 | 18 | |  |
| Navarro, L. unpublished data | | *Lantana camara* | | Verbenaceae | | Fruit set | | 42°52'N // 8°38'W | | 0.247 ± 0.035 (7) | | 0.046 ± 0.053 (7) | | -4.188 | 0.912 | 14 | |  |
| Navarro, L. unpublished data | | *Linaria triornitophora* | | Scrophulariaceae | | Fruit set | | 42°52'N // 8°38'W | | 0.624 ± 0.254 (8) | | 0.591 ± 0.144 (8) | | -0.151 | 0.251 | 16 | |  |
| Navarro, L. unpublished data | | *Linaria vulgaris* | | Scrophulariaceae | | Fruit set | | 42°52'N // 8°38'W | | 0.801 ± 0.245 (7) | | 0.597 ± 0.109 (9) | | -1.069 | 0.290 | 16 | |  |
| Navarro, L. unpublished data | | *Lithodora prostrata* | | Boraginaceae | | Fruit set | | 42°53'N // 8°28'W | | 0.240 ± 0.050 (32) | | 0.180 ± 0.070 (27) | | -0.988 | 0.077 | 59 | |  |
| Navarro, L. unpublished data | | *Lonicera periclymenum* | | Caprifoliaceae | | Fruit set | | 42°38'N // 8°48'W | | 0.170 ± 0.130 (23) | | 0.140 ± 0.060 (20) | | -0.284 | 0.094 | 43 | |  |
| Navarro, L. unpublished data | | *Macleania stricta* | | Ericaceae | | Fruit set | | 1°13'N // 77°59'W | | 0.287 ± 0.087 (12) | | 0.116 ± 0.055 (12) | | -2.276 | 0.275 | 24 | |  |
| Navarro, L. unpublished data | | *Melampyrum nemorosum* | | Orobanchaceae | | Fruit set | | 54° 5'N // 24°15'E | | 0.646 ± 0.270 (7) | | 0.577 ± 0.266 (8) | | -0.242 | 0.270 | 15 | |  |
| Navarro, L. unpublished data | | *Melampyrum polonicum* | | Orobanchaceae | | Fruit set | | 54° 5'N // 24°15'E | | 0.591 ± 0.358 (9) | | 0.537 ± 0.460 (9) | | -0.124 | 0.223 | 18 | |  |
| Navarro, L. unpublished data | | *Melampyrum pratense* | | Orobanchaceae | | Fruit set | | 42°38'N // 7° 7'W | | 0.485 ± 0.215 (10) | | 0.307 ± 0.245 (9) | | -0.738 | 0.225 | 19 | |  |
| Arroyo, J. unpublished data | | *Narcissus papyraceus* | | Amaryllidaceae | | Fruit set | | 36° 8'N // 5°41'W | | 0.201 ± 0.131 (22) | | 0.062 ± 0.059 (22) | | -1.344 | 0.111 | 44 | |  |
| Navarro, L. unpublished data | | *Nicotiana glauca* | | Solanaceae | | Fruit set | | 28°22'N // 16°52'W | | 0.880 ± 0.086 (6) | | 0.627 ± 0.193 (6) | | -1.559 | 0.435 | 12 | |  |
| Navarro, L. unpublished data | | *Odontonema strictum* | | Acanthaceae | | Fruit set | | 18°24'N // 66° 2'W | | 0.543 ± 0.210 (10) | | 0.118 ± 0.045 (8) | | -2.526 | 0.402 | 18 | |  |
| Navarro, L. unpublished data | | *Palicourea croceoides* | | Rubiaceae | | Fruit set | | 18°19'N // 65°43'W | | 0.460 ± 0.143 (10) | | 0.384 ± 0.178 (10) | | -0.451 | 0.205 | 20 | |  |
| Navarro, L. unpublished data | | *Passiflora mixta* | | Passifloraceae | | Fruit set | | 0°54'S // 78°43'W | | 0.283 ± 0.191 (4) | | 0.162 ± 0.084 (5) | | -0.762 | 0.482 | 9 | |  |
| Navarro, L. unpublished data | | *Pedicularis sylvatica* | | Scrophulariaceae | | Fruit set | | 42°38'N // 7° 7'W | | 0.421 ± 0.098 (15) | | 0.296 ± 0.079 (15) | | -1.359 | 0.164 | 30 | |  |
| Navarro, L. unpublished data | | *Ruellia tuberosa* | | Acanthaceae | | Fruit set | | 18°20'N // 65°43'W | | 0.542 ± 0.099 (8) | | 0.496 ± 0.241 (7) | | -0.244 | 0.270 | 15 | |  |
| Navarro, L. unpublished data | | *Russelia equisetiformis* | | Scrophulariaceae | | Fruit set | | 18°24'N // 66° 2'W | | 0.591 ± 0.247 (10) | | 0.540 ± 0.229 (10) | | -0.205 | 0.201 | 20 | |  |
| Navarro, L. unpublished data | | *Salvia haenkei* | | Lamiaceae | | Fruit set | | 16°23'S // 71°31'W | | 0.834 ± 0.016 (5) | | 0.792 ± 0.074 (5) | | -0.713 | 0.425 | 10 | |  |
| Navarro, L. unpublished data | | *Salvia verbenaca* | | Lamiaceae | | Fruit set | | 42°29'N // 6°50'W | | 0.851 ± 0.080 (11) | | 0.765 ± 0.118 (10) | | -0.817 | 0.207 | 21 | |  |
| Navarro, L. unpublished data | | *Siphocampylus aureus* | | Campanulaceae | | Fruit set | | 10°25'N // 84° 1'W | | 0.406 ± 0.241 (8) | | 0.287 ± 0.278 (7) | | -0.432 | 0.274 | 15 | |  |
| Navarro, L. unpublished data | | *Siphocampylus aureus* | | Campanulaceae | | Fruit set | | 8°36'N // 71° 7'W | | 0.439 ± 0.179 (6) | | 0.358 ± 0.263 (4) | | -0.339 | 0.422 | 10 | |  |
| Navarro, L. unpublished data | | *Sphyrospermun sp.* | | Ericaceae | | Fruit set | | 1°13'N // 77°59'W | | 0.310 ± 0.077 (6) | | 0.156 ± 0.123 (6) | | -1.382 | 0.413 | 12 | |  |
| Navarro, L. unpublished data | | *Stachytarpheta jamaicensis* | | Verbenaceae | | Fruit set | | 21°53'N // 84°42'W | | 0.576 ± 0.178 (14) | | 0.529 ± 0.160 (13) | | -0.269 | 0.150 | 27 | |  |
| Navarro, L. unpublished data | | *Thunbergia grandiflora* | | Acanthaceae | | Fruit set | | 18°21'N // 65°38'W | | 0.540 ± 0.110 (5) | | 0.526 ± 0.074 (5) | | -0.132 | 0.401 | 10 | |  |
| Navarro, L. unpublished data | | *Thunbergia grandiflora* | | Acanthaceae | | Fruit set | | 43°40'N // 7°23'W | | 0.463 ± 0.110 (6) | | 0.349 ± 0.214 (6) | | -0.617 | 0.349 | 12 | |  |
| Navarro, L. unpublished data | | *Trifolium campestre* | | Fabacecae | | Fruit set | | 42°29'N // 6°50'W | | 0.496 ± 0.185 (21) | | 0.535 ± 0.194 (21) | | 0.199 | 0.096 | 42 | |  |
| Navarro, L. unpublished data | | *Weigela florida* | | Caprifoliaceae | | Fruit set | | 42°52'N // 8°38'W | | 0.713 ± 0.120 (15) | | 0.090 ± 0.099 (15) | | -5.483 | 0.634 | 30 | |  |
| Navarro, L. unpublished data | | *Wisteria sinensis* | | Fabacecae | | Fruit set | | 42°52'N // 8°38'W | | 0.691 ± 0.070 (11) | | 0.117 ± 0.068 (11) | | -8.033 | 1.648 | 22 | |  |
| Navarro, L. unpublished data | | *Duranta erecta* | | Verbenaceae | | Fruit set | | 22°36'N // 83°41'W | | 0.771 ± 0.306 (11) | | 0.210 ± 0.115 (11) | | -2.334 | 0.306 | 22 | |  |
